# Genomic ancestry and diversity in individuals with type 1 diabetes from Brazil and Portugal: a descriptive analysis

**DOI:** 10.1186/s13098-026-02144-w

**Published:** 2026-05-10

**Authors:** Lívia Leite Ferreira, Sónia do Vale, Miguel António Duarte, Ana Luísa Silva, Luís Cristóvão Porto, Andreia C. Turchetto-Zolet, Dayse A. Silva, Marília Brito Gomes

**Affiliations:** 1 DNA Diagnostic Laboratory, Rio de Janeiro State University, Rua São Francisco Xavier, Rio de Janeiro, RJ 524, 20550-900 Brazil; 2 Postgraduate Program in Genetics and Molecular Biology, Department of Genetics, Institute of Biosciences, Federal University of Rio Grande Do Sul (UFRGS), Porto Alegre, Rio Grande Do Sul Brazil; 3 Diabetes Unit, State University of Rio de Janeiro, Rio de Janeiro, RJ Brazil; 4 Histocompatibility and Cryopreservation Laboratory (HLA), Rio de Janeiro State University (UERJ), Rio de Janeiro, Brazil; 5 Endocrinology department, Unidade Local de Saúde Santa Maria, Lisbon, Portugal; 6 Faculdade de Medicina, Universidade de Lisboa, Lisbon, Portugal; 7 Faculdade de Medicina, Instituto de Saúde Ambiental (ISAMB), Universidade de Lisbon, Lisbon, Portugal

**Keywords:** Genomic ancestry and diversity, Type I diabetes, Portugal and Brazil

## Abstract

**Introduction:**

Type 1 diabetes (T1D) is a chronic autoimmune disease with variable incidence worldwide,. with a higher incidence in countries of European ancestry. The present study aimed primarily to evaluate differences in genomic ancestry (GA) and genetic diversity (GD) between individuals with T1D in Brazil and Portugal compared with healthy control. Secondarily, we aimed to compare the GA and to compare the GA of individuals with T1D from different regions of Brazil with those from Portugal.

**Methods:**

The sample included 1,698 Brazilians and 107 Portuguese individuals with T1D, whose data were analyzed using Ancestry Informative Markers (46-AIMs-Indels) to estimate the proportion of European, African, and Native American (NAM) ancestry. Allele frequencies for short (1) and long (2) alleles were estimated for all individuals. Fragment genotyping was performed by capillary electrophoresis using the ABI 3500 sequencer (Applied Biosystems). The data were analyzed using GeneMapper V.4.1 (Life Technologies, USA). STRUCTURE v2.3.3 software was used to estimate individual ancestry, with K values ranging from 2 to 5 to assess the robustness of the inferred ancestry proportions based on the HGDP-CEPH diversity panel (H952 subset) as a reference for ancestral populations. Genetic parameters were estimated using the software Arlequin v3.5.2.2

**Results:**

The results revealed a predominance of European GA in both populations, with higher European GA in Portugal and higher African and NAM GA in Brazil. A higher GD was observed in individuals with T1D from Brazil compared with those from Portugal. Non-admixed individuals with T1D from Portugal exhibited high genetic homogeneity and low genetic diversity. Individuals with T1D from the Northern Region of Brazil presented the greatest genetic differentiation compared to regional control groups..

**Conclusion:**

This study identifies differences in GA and GD among individuals with T1D from Brazil and Portugal. Considering the dynamics of migrations and admixture in both countries, our data should drive future research areas related to identifying other genetic variants, such as the HLA system alleles (risk or protection) in Brazil and Portugal, which may contribute to a better understanding of the pathogenesis of the disease in both countries.

**Supplementary Information:**

The online version contains supplementary material available at 10.1186/s13098-026-02144-w.

## Introduction

Type 1 diabetes mellitus (T1D) is one of the most common chronic autoimmune diseases in childhood and adolescence. It is characterized by the destruction of pancreatic beta cells, leading to insulin deficiency, hyperglycemia, and lifelong dependence on exogenous insulin [1, 2]. Etiology involves a complex interaction between genetic and environmental factors, while demographic data and economic and health indicators of populations can also impact the variability in T1D incidence worldwide [3–6]

According to the Type 1 Diabetes Index, 8.7 million people worldwide live with T1D (T1D Index, 2024). Globally, more than 78,000 youths are diagnosed with T1D each year, with incidence rates varying across countries [3, 7, 8]. Nowadays, East Asians and Native Americans exhibit the lowest incidence rates (0.2–8 per 100,000 people/year), while Northern European countries have the highest rates, with Finland reporting the highest global rate (> 64.2 per 100,000 people/year) [3, 9].

In Portugal, in 2023, the incidence of T1D was 20/100.000 in individuals aged ≤ 14 years [10]. Although in Brazil, a continental country, it was estimated an overall incidence of 7.6 per 100,000 people/year (IDF, 2022), in Bauru, a Southeast Brazilian city, a higher incidence of 12.8/100,000 in individuals aged ≤ 14 years was noted in 2015 [11], reflecting that there may be a large variability in the incidence of the disease in the country.

Like many countries in South America, the Brazilian population results from recent migratory flows and genetic admixture between different ethnic groups [12, 13]. The Native Americans who lived in Brazil evolved in isolation until the sixteenth century [14–16]. Contact with Europeans, especially the Portuguese and Spanish, along with wars, diseases, and reproductive isolation, significantly impacted the genetic composition of native populations [15, 16]. Later, the slave forced entry of Africans into the Americas introduced a third ethnic component [15, 16]. Thus, Brazil has significant GD and is more likely to possess a population substructure.

Conversely, Portugal, one of the countries from which the Brazilian migratory population originated, presents a more homogeneous genetic composition. It also had been inhabited by numerous peoples before (Roman, Byzantine domination; Visigothic; Islamic presence, Christians, Jews, and Berbers from North Africa (Paim, 2000; Aréan-Garcia, 2009; Garcia, 2009; Fausto, 2012). [17]. The population that formed there over the following eight centuries consisted of a complex demographic who coexisted amidst diverse political landscapes, economic strategies, religious beliefs, and population movements [17, 18] Later, as a major economic strategy, the Kingdom of Portugal developed maritime trade routes to Africa, northern Europe, and South America, marking the beginning of the colonization of the Americas [19].

In this context, Ancestry Informative Markers (AIMs) are genetic tools used to estimate the GA composition of individuals and populations [12]. These markers exhibit significant intercontinental allele differentiation, making them particularly useful for estimating proportions of genetic admixture in populations such as Brazil [20]. In Brazil, the use of AIMs has advanced the understanding of the relationship between ancestry and disease predisposition, providing insights into the impact of genetic admixture on susceptibility patterns to some diseases, such as sickle cell anemia, nephropathy, and fatty liver disease [21–28] reported that the genetic profile of individuals with T1D in Brazil exhibited a higher median of individual European ancestry [28].

Thus, considering the intertwined history between the two countries due to colonization, this study aimed primarily to evaluate the GA and GD difference between the individuals with T1D in Brazil and Portugal through the proportions of European, African, and NAM GA. Afterwards, we compared them with healthy individual controls from the HGDP-CEPH reference panel [29] and the Brazilian reference panel [20]. Secondarily, we intend to investigate the GA of individuals with T1D from different regions of Brazil and from Portugal. Identifying the differences in GA and GD composition between the two populations could improve understanding of ancestry structure in individuals with T1D in both countries.

## Methodology

### Individuals with T1D

This work is part of a multicenter study on type 1 diabetes carried out between August 2011 and August 2014 Written informed consent was obtained from all participants or their parents. A standardized questionnaire was also administered during a clinical visit to collect demographic and clinical data, including gender, current age, birthplace, self-reported race/ethnicity, age at diagnosis, and duration of diabetes. The Brazilian T1D samples analyzed in this study were previously described by [28]. A standardized questionnaire was also administered during a clinical visit to assess clinical and demographic data, including current age, age at diagnosis, self-reported color-race (White, Black, Brown (“parda”), Asian (“amarela”), and Native American (“indígena”)), [30] diabetes duration and place of birth of these individuals, their parents, and grandparents. Participants were included who had a minimum of six months of medical follow-up at the respective center. T1D was diagnosed by a physician based on the classical clinical presentation (polyuria, weight loss, polydipsia, and continuous insulin use since then). Those who did not meet the inclusion criteria were excluded (pregnant or lactating women, and those with an acute infection or ketoacidosis within the 3 months prior to enrollment).

The samples from Portuguese individuals were collected at Hospital Santa Maria in Lisbon, as part of a study on type 1 diabetes involving residents of the south region of Portugal (Lisbon and Tagus Valley, Alentejo, and Algarve). All 107 Portuguese individuals with T1D reside in Portugal, of whom 93 are of entirely Portuguese ancestry, and 14 are of admixed ancestry. Additionally, the countries of birth of these individuals, their parents, and their grandparents were also recorded, representing Portugal, China, São Tomé and Príncipe, Cape Verde, Angola, Mozambique, Guinea-Bissau, the Democratic Republic of Congo, Spain, France, Brazil, and Argentina.

This study was approved by the ethics committee of the Lisbon Academic Medical Center (CAAE Nº15/18), affiliated with Hospital Santa Maria, and the Research Ethics Committee of the HUPE-UERJ (Pedro Ernesto University Hospital, Rio de Janeiro State University), with ethical approval and the local ethics committee at each center, with the number CAAE 53563115.2.1001.5259. The standardized questionnaire was also administered during clinical consultations to assess clinical and demographic data as described previously. All procedures performed were in accordance with the ethical standards of the Helsinki Declaration of 1964 and later versions. Informed consent was obtained from all patients before recruitment.

### Amplification and genotyping

Allele frequencies for short (1) and long (2) alleles were estimated for 1698 Brazilian patients and 107 Portuguese patients (Supplementary Table 2). The global GA data for Brazilian patients with type 1 diabetes were previously reported by [28]. For the GA data of patients with type 1 diabetes residing in Portugal, PCR amplification also used the same panel of 46-AIM-INDELs, as outlined in the standardized protocol described by [29]. Fragment genotyping was performed by capillary electrophoresis using the ABI 3500 sequencer (Applied Biosystems). The data were analyzed using GeneMapper V.4.1 (Life Technologies, USA). Rigorous quality control (QC) procedures were applied to ensure genotyping accuracy and data reliability. QC steps included visual inspection of all electropherogram peaks and evaluation of genotype clustering quality in GeneMapper. Markers or samples that failed to meet the quality control criteria were subjected to a new PCR reaction for the marker in question to obtain high-quality data. Structure V.2.3.3 software (K values ranging from 2 to 5 to assess the robustness of the inferred ancestry proportions) was used to estimate individual ancestry based on the HGDP-CEPH diversity panel (H952 subset) as a reference for ancestral populations. For contextual comparison with the Brazilian background population, we used the dataset from [20], which includes non-T1D individuals sampled from the same Brazilian geographic regions represented in our cohort.

### Statistical analyses

The genetic parameters of the two populations (genetic differentiation, Hardy–Weinberg equilibrium, genetic diversity, and allele frequencies) were estimated using the software Arlequin v3.5.2.2 [30]. Brazilian individuals with T1D were grouped by place of birth (Midwest, Southeast, South, North, and Northeast) and stratified into non-admixed if they had any ancestry > 90% [31], otherwise, they were classified as admixed. Portuguese individuals with type 1 diabetes were stratified into non-admixed if they had GA greater than 90% [31] otherwise, they were classified as admixed one.

For Fst analysis, were computed in Arlequin v3.5 using the Weir & Cockerham estimator. Statistical significance was assessed using 10,000 permutations. A full pairwise FST matrix (with corresponding p-values) is provided in Supplementary Table S1. Brazilian population was divided into groups based on the geographic region of birth of each patient (North, Northeast, South, Southeast, and Midwest), while the Portuguese individuals with T1D were categorized according to their ancestral background (Portuguese non-admixed or admixed-ancestry).

According to [32], Fst values within the range 0–0.05 indicate low genetic differentiation, 0.05–0.15 moderate differentiation, 0.15–0.25 high differentiation, and values above 0.25 as very high genetic differentiation.

Using STATISTICA v.13 (13.2 Trial; StatSoftInc., http://www.statsoft.com.br) [46], a multidimensional scaling (MDS)[48] scatter plot was constructed using the paired Fst matrix (stress = 0.0219701). The groups mentioned above, along with subsets of European (Orkney Islands; Russia-Caucasus (Adygei and Russian); France (Basque, and French); Italy (Bergamo, Sardinian, and Tuscan), African (Bantu- South Africa—Lesotho; Botswana Or Namibia; Angola- Kenya (Bantu); Senegal (Mandenka); Nigeria (Yoruba); Central African Republic (BiakaPygmy); Congo (MbutiPygmy); Namibia (San); and Native Americans (Colombia (Colombian); Brazil (Karitiana and Surui); Mexico (Maya and Pima) populations from the HGDP-CEPH and a Brazilian control population [20] and were included in the analysis. These reference groups capture the major continental ancestral components underlying the genetic background of contemporary Brazilians, as well as the two primary ancestry contributions to Portuguese populations (European and African).

Demographic data, including place of birth, sex, age, disease duration, GA, and self-reported color-race, were analyzed using IBM SPSS Statistics (Version 17) [47]. For comparisons between countries and between GA and T1D, age, and duration of T1D, the nonparametric Mann–Whitney test was used. For categorical variables, the Chi-square test was performed. Continuous variables were presented as means (± SD). A subsequent analysis compared patients who were non-admixed with those who were admixed in each country. A p-value < 0.05 was considered significant for all comparisons and analyses.

## Results

### Overview of demographic data of the studied population (Brazil and Portugal)

The study included 1,436 admixed and 262 non-admixed Brazilian individuals with T1D, as well as 14 admixed and 93 non-admixed Portuguese. The demographic, clinical, and GA ancestry data of the sample and the comparison among the groups are detailed in Table 1. Portuguese individuals with T1D (general, non-admixed, and admixed) exhibited a higher age and duration of diabetes than Brazilian individuals with T1D (general, non-admixed, and admixed). No difference in the distribution by sex was observed.

**Table 1:** Clinical, demographic and genomic ancestry data of studied population

**Variable**	**T1D (Brazil)**	**T1D Portugal**	**p-value**	**T1D Brazil non AD**	**T1D Portugal non AD**	**p-value**	**T1D Brazil AD**	**T1D Portugal AD**	**p-value**
N, (%)	1,698	107		262(14.5)	93(86.9)		1436(79.6)	14(0.8)	
Sex, male (n, %)	753 (44.3)	45 (42.1)	0.6	130(49.6)	40 (43.0)		813(56.6)	5 (35.7)	0.56
Age, y	30.1 ± 11.9	42.5 ± 14.2	<0.001	31.8 ± 12.0	42.5 ± 14.2		29.7 ±11.9	39.1 ± 17.1	<0.001
Age at diagnosis, y	15.00 ± 9.2	19.6 ± 12.1	<0.001						
Duration of T1D, y	15.5±9.3	22.9±14.2	**<**0.001	16.9±9.9	23.8±14.1		15.2±9.1	16.3±13.3	ns
**Self-reported color-race (n,%)**			<0.001						<0.001
White	923 (54.4)	95 (88.8)		201(76.7)	93 (100)		722 (50.3)	2 (14.3)	
Black	132 (7.8)	9 (8.4)		2(0.8)	0		130 (9.1)	9 (64.3)	
Brown	610 (35.9)	3 (2.8)		58(22.1)	0		552 (38.4)	3 (21.4)	
Asian	18(1.1)	0		1(0.4)	0		17(1.2)	0	
Native American	15 (0.9)	0		0	0		15(1.0)	0	
**Geographic Region**									
Southeast	803 (44.5)	0							
Northeast	465 (25.8)	0							
South	231(12.8)	0							
Midwest Region	154 (8.5)	107 (100)							
North	45 (2.7)	0							
GA (%)									
European	66.4±20.6	88.9±25.1	**<**0.001	94.2 ± 2.3	97.48±3.0	**<**0.001	61.3±18.2	32.62 ±33.86	0.007
**Minimum/** **Maximum**	2.3to99.1	0.3 to 99.6							
**Mean Difference (CI)**			−22.6(−26.9;−18.5)						
African	19.9±16.8	9.2±22.9	<0.001	2.7±1.8	1.43±2.24	<0.001	23.1±16.4	60.54±31.49	0.001
**Minimum/** **Maximum**	0.2;88.7	0.2;99.5							
**Mean difference (CI)**			10.8(7.4;14)						
Native American	13.6±11.5	1.8±5.8	**<**0.001	3.1±1.9	01.09±2.11	**<**0.001	15.5±11.4	6.85±14.66	0.045
**Minimum/** **Maximum**	0.30;69.5	0.20;49.5							
**Mean difference (CI**			11.8(9.5;13.)						

Data are presented as mean (± SD); n = number (%); T1D, = type 1 diabetes; GA = Genomic Ancestry; n = number; AD = admixed (applies to individuals in whom some of the ancestral components reached >90%).;^*^ < 0.001 vs T1D Brazil (non AD and AD);^*^ < 0.001 vs all the other groups. Data are presented as mean (± SD). No deviation from Hardy-Weinberg equilibrium was observed after Bonferroni correction (p < 0.001). Brazil ad x Portugal not-Ad:

Self-reported color-race in the overall sample indicated that more Brazilian individuals with T1D self-reported as Brown and more Portuguese self-reported as White. Considering the admixed individuals with T1D, Brazil had more White and Brown individuals, and Portugal had more Black individuals with T1D. Among Portuguese non-admixed individuals with T1D, 100% self-reported as White and none self-reported as Black, Brown, Asian, or Native American.

Regarding birthplace, Brazilian individuals with T1D were distributed according to geographic regions of Brazil: Southeast (48.5%), Northeast (29.2%), South (10.9%), Midwest (8.9%), and North (2.6%). All Portuguese individuals with T1D were from Central Portugal (Lisbon).

### Overview of genetic data of the studied population (Brazil and Portugal)

**Fig. 1:**
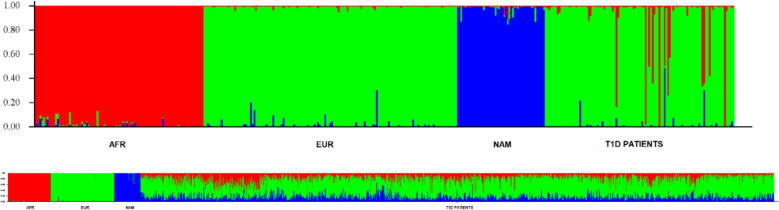
Individual ancestry estimates obtained for the HGDP-CEPH reference samples and for individuals with T1D from Portugal and Brazil [28] using 46 AIM-INDELs. The STRUCTURE results (K = 3) show clear contrasts in continental ancestry: Portuguese participants display predominantly European ancestry, while Brazilian participants show a more variable genomic composition with higher African and Native American contributions. AFR: African; EUR: European; NAM: Native American. Ancestry estimates were obtained using STRUCTURE, for the following options: k = 3; 100,000 burning steps followed by100,000 MCMC iterations; Admixture model (“Use population Information to test for migrants”); and allele frequencies were correlated and updated using only individuals with POPFLAG = 1

**Fig. 2:**
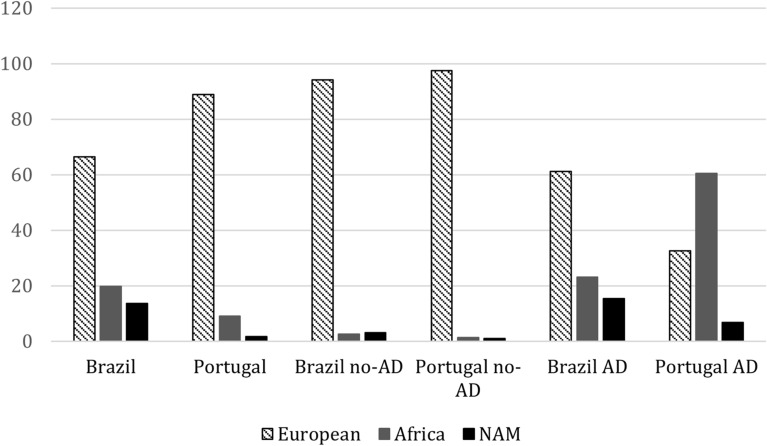
Mean genomic ancestry proportions (European, African, and NAM) observed in the six groups analyzed: individuals with T1D from Brazil, Portugal, no-admixed Brazilians (> 90% single-continental ancestry), no-admixed Portuguese, admixed Brazilians, and admixed Portuguese. Values represent average ancestry estimates derived from the 46 AIM-INDEL panel

The mean GA for each group is described in Table 1, Fig. 1 and Fig. 2 Considering the overall sample, Brazilian individuals with T1D had lower European GA and higher African and NAM GA than Portuguese individuals with T1D. A higher European GA was observed in Portuguese non-admixed individuals with T1D compared with non-admixed individuals with T1D from Brazil. Brazilian non-admixed individuals with T1D had a higher African and NAM GA than Portuguese non-admixed individuals with T1D. More non-admixed individuals with T1D from Portugal had a European GA > 90% than those from Brazil, p < 0.001. Among the overall sample (Brazil and Portugal), only three admixed individuals with T1D from Portugal had African GA > 90%, and none had NAM GA > 90%. A higher African GA was observed in admixed individuals with T1D from Portugal than in those from Brazil. A higher European and NAM GA was observed in admixed individuals with T1D from Brazil than in those from Portugal.

### Overview of genetic differentiation data of the studied population (Brazil and Portugal)

Data from the Brazilian and Portuguese populations (control and T1D individuals) were used to estimate genetic differentiation using Fst (Supplementary Table 1). Given the properties of the AIM-INDEL panel, these results are expected, as the markers were specifically selected to maximize differentiation among continental ancestry components rather than to capture fine-scale population structure.

The overall sample of Brazilian individuals with T1D exhibited low genetic differentiation compared to those with T1D from each Brazilian geographic region (Fst 0 to 0.05) and compared to those individuals with T1D from Portugal (Fst 0.05–0.15) and to European control individuals (0–0.05). All p-values for the Fst data were significant (p < 0.01). The other comparisons among individuals with T1D from different regions of Brazil, as well as comparisons between individuals with T1D from Brazil and Portugal, and between control individuals from Brazil and Europe, with their respective levels of significance and genetic differentiation, are described in Supplementary Table 1.

### Overview of GD data of the studied population (Brazil and Portugal)

Table 2 shows the GD observed within each group (controls and diabetes) for the 46 AIM-INDELs. Because these markers are selected for high intercontinental differentiation, these values reflect diversity within this specific panel rather than general genomic diversity. Therefore, the results are presented descriptively and should not be interpreted as overall population genetic diversity. The diversity index observed in the Brazilian groups was 0.404651 ± 0.198326, and Portugal was observed to have a 0.350528 ± 0.173502. Considering miscegenation as a factor that increases this diversity for both groups of individuals with type 1 diabetes.

**Table 2:** Genetic diversity in the studied population

Locatiton	Reference	Number of individuals	Number of haplotypes	Gene diversity over loci
Brazil T1D	This study	1698	3053	0.404651 ± 0.198326
Portugal T1D	This study	107	212	0.350528 ± 0.173502
T1D Brazil Non AD	This study	262	446	0.364625 ± 0.179777
T1D Portugal Non AD	This study	93	186	0.330584 ± 0.164149
T1D Brazil AD	This study	1436	2806	0.409177 ± 0.200492
T1D Portugal AD	This study	14	28	0.386876 ± 0.196529
Midwest T1D	This study	154	89	0.398670 ± 0.197500
North T1D	This study	45	90	0.395636 ± 0.196054
Northeast T1D	This study	465	918	0.418531 ± 0.205041
South T1D	This study	231	462	0.384759 ± 0.189283
Southeast T1D	This study	803	1594	0.405082 ± 0.198608
North Control	[20]	42	280	0.343438 ± 0.169964
Northeast Control	[20]	237	469	0.403862 ± 0.198468
Midwest Control	[20]	84	252	0.403745 ± 0.198753
Southeast Control	[20]	509	1014	0.391642 ± 0.192327
South Control	[20]	64	127	0.365946 ± 0.181338
African Control	[29]	105	210	0.288534 ± 0.144129
Europe Control	[29]	158	316	0.322024 ± 0.159933
Native American Control	[29]	64	128	0.270041 ± 0.135746

**Fig. 3:**
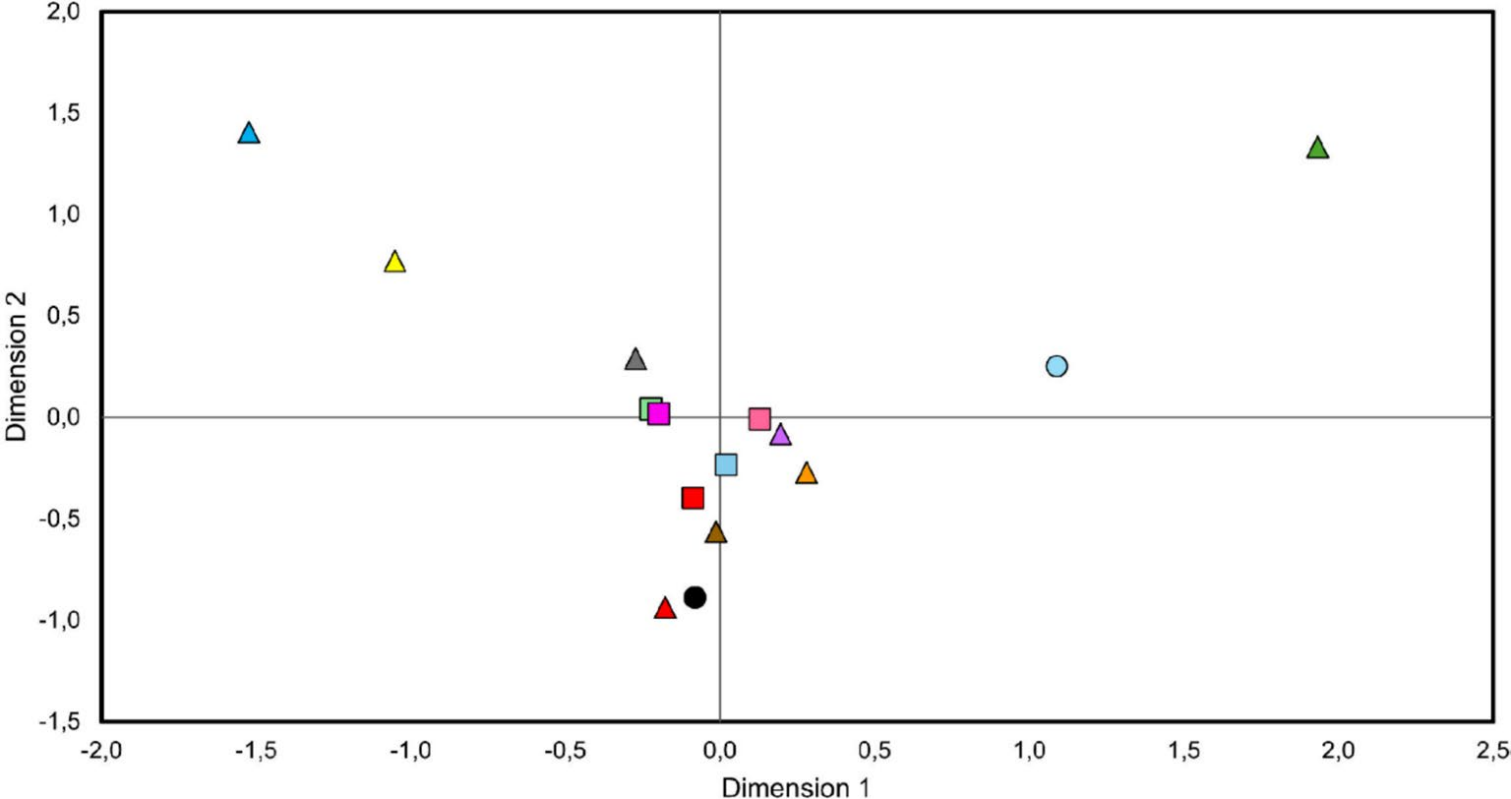
Multidimensional Scaling (MDS) plot based on pairwise FST values. Triangles represent the control populations [20, 29], circles represent the population with diabetes from Portugal, and squares represent the population with diabetes from Brazil, categorized by their geographic region of birth The plot illustrates the genetic proximity among groups: non-admixed Portuguese T1D individuals cluster closely with European controls, whereas admixed Portuguese individuals shift toward African controls. Brazilian T1D groups distribute along the European–African–Native American gradient, mirroring Brazil’s heterogeneous admixture patterns. The low stress value (0.0219) supports the reliability of the two-dimensional representation.”. Native Control;  African Control; Europe Control;  North Control;  Midwest Control;  Northeast Control; Southeast Control;  South Control;  Portugal non-admixed;  Portugal admixed; Midwest T1D; North T1D; Northeast T1D; Southeast T1D; South T1D

To visually represent the Fst matrix, a multidimensional scaling (MDS) plot was created (Fig. 3). MDS is a tool that enables researchers to obtain quantitative estimates of similarity between groups of items [32]. Admixed Portuguese individuals with T1D are more similar to the African control population and the Brazilian Northeast individuals with T1D. On the other hand, the non-admixed individuals with T1D exhibit greater similarity to the European Control and Brazilian Southern Control populations. The remaining individuals with T1D clustered closer to the control groups from each Brazilian region.

## Discussion

To our knowledge, this study is the first to address individuals with T1D in two countries with an intertwined history for over 500 years and to present data obtained through the analysis of AIMs-INDELs to estimate European, African, and NAM GA. As expected, non-admixed Portuguese individuals with T1D have higher European GA and very low African and NAM GA, and are more homogeneous than Brazilian individuals with T1D. Admixed individuals with T1D from Portugal who were mainly of African origin showed greater African GA than to individuals with T1D from Brazil. A higher diversity within the 46 AIM-INDELs panel in the overall Brazilian sample, which can be explained by the high African and NAM GA contribution. These findings also highlight the need for broader characterization of AIM-INDEL diversity in contemporary Brazilian populations, as current knowledge of their distribution is limited.

Based on Fst analysis, the groups compared showed low genetic differentiation, including an overall comparison between Brazilian and Portuguese individuals with T1D. The genetic differentiation between individuals with T1D from Brazil and non-admixed Portuguese individuals with T1D was lower than that from admixed Portuguese individuals with T1D, which is probably related to the predominance of European GA in Brazil. Except for individuals with T1D from the Brazilian Northern Region, characterized by a high proportion of indigenous people, all other groups studied showed greater genetic differentiation than to the NAM control individuals. Exception for admixed individuals with T1D from Portugal, characterized by the highest African GA, all other groups studied showed high and very high genetic differentiation compared to the African control individuals. Although it is not within the scope of the present study, this latter fact may reflect differences in ancestry proportions captured by the 46-AIMs-INDELs panel used in the present study.

Considering individuals with T1D in Brazil, the Northern Region presented the greatest genetic differentiation compared to control individuals from each region of the country. In contrast, individuals with T1D from the Southern Region showed the lowest genetic differentiation compared to non-admixed individuals with T1D from Portugal.

Given the low, albeit significant, genetic differentiation observed between Brazil and Portugal, it is inferred that the European Caucasian alleles found in the Brazilian population could be primarily inherited from Portuguese individuals during colonization or during the period of the policy known as the “Whitening of Brazil” when Italian, Portuguese, and Spanish individuals have immigrated to Brazil [33]. Consequently, the contributions of these alleles may reflect historical ancestry contributions to the Brazilian population,however, no inference about T1D risk can be made from these data. However, it is important to emphasize that previous study by our group did not show a relationship between European genomic ancestry and age at diagnosis in Brazilian individuals with T1D [34]. While we cannot rule out the potential contribution of NAM and African genetic pools to individuals with T1D in Brazil, the higher European GA in Brazilian T1D than in African and NAM populations suggests a greater influence of European ancestry. However, we still lack knowledge regarding which alleles could be involved in both genetic pools.

As shown in Table 2, the GD observed in the Brazilian population was higher than that in the Portuguese population across the three comparisons: Brazil vs. Portugal; admixed Brazilians vs. admixed Portuguese; and non-admixed Brazilians vs. non-admixed Portuguese. This outcome was expected given the properties of the markers used, which were selected for maximal intercontinental differentiation. This difference reflects the distinct demographic histories of the two populations. The Portuguese population has a largely homogeneous origin shaped by thousands of years of internal admixture, as previously mentioned. In contrast, the Brazilian population derives from a tri-hybrid contribution of distinct groups: between 1500 and 1972, 58% of immigrants were Europeans, 40% were Africans, and 2% were Asians, in addition to the Indigenous peoples who already inhabited the territory [35–37].

This association between European GA and diseases has been observed in other studies, such as the work by [38], which reports a higher proportion of European GA in Brazilian individuals with multiple sclerosis, an autoimmune disease. However, for non-alcoholic fatty liver disease, [21] report it to be more prevalent among individuals with the highest African GA. As we did not investigate other genetic or immunological markers related to T1D risk/protection, it is important to emphasize that our data do not allow etiologic inference for T1D.

As previously reported [20], Brazilian control individuals from the North Region exhibit a higher NAM composition than those from other regions. However, as observed in Fig. 3, individuals with T1D from the North are somewhat distant from the Native American control individuals, due to their higher proportion of European GA, as previously reported [28]. Conversely, individuals in the South region with T1D show the highest European GA, similar to that of their control individuals. Meanwhile, as reported by [20], the Southeast and Northeast Regions exhibit an admixture of the three ancestries, positioning them between control populations of European, Native American, and African origin. It is noteworthy that the genetic differentiations among Brazilian regions, despite some differences, are generally low but statistically significant. It is also important to highlight, as stated by Gomes, that when comparing the frequency of ancestries between individuals with T1D and the control group in Brazil, across all geographic regions, the proportion of European GA was significantly higher in the individuals with T1D [28].

Non-admixed Portuguese individuals with T1D also showed GA estimates within the expected range, similar to those of European control individuals. This was expected, as nearly the entire group exhibited European GA > 90%.

Similarly, admixed Portuguese individuals with T1D exhibited a higher African contribution than non-admixed individuals with T1D. Both the Portuguese and other European populations have resulted from an ancient and gradual process of admixture with different populations and gene flows in historical times, which have played a crucial role in shaping their current GD patterns [35, 36].

Due to its geographical proximity to Africa [35], the Portuguese population had already experienced admixture with African populations before the colonization of Brazil. An example of this is the population of Cape Verde, one of the first admixed populations, originating in the fifteenth century from the contact between Portuguese and West African populations in a previously uninhabited archipelago off the West African coast [39, 40]. Also, the previous colonization of African countries such as Angola and Mozambique), the decolonization in the twentieth century, and more recent migrations between those countries and Portugal, namely immigration from African Countries of Portuguese Official Language (ACPOL), have promoted admixed populations [41]. This explains the high frequency of African ancestry among admixed Portuguese individuals with T1D. Additionally, in the admixed group, African GA exceeds 90% in three individuals, with an average of 60%, suggesting that admixture in this population could be recent [31].

In contrast to the Portuguese population, the Brazilian population exhibits a complex structure resulting from admixture among three distinct genetic components: European, Native American, and African [20, 42]. Initially, the Brazilian population comprised approximately 2.5 million Native Americans [43]. With the arrival of European colonists, primarily men, admixture began in Brazil, occurring asymmetrically under the colonial model, in which Portuguese men extensively admixed with Native American women and later with African women [33]. Our data indicate a low frequency of NAM in individuals with T1D, which is explained by the dramatic population bottleneck Native Americans experienced during colonization [31].

Studying ancestry in admixed populations is crucial since continental populations differ significantly in disease predispositions due to local adaptations and selective factors [33]. As reported by Vaulin & cols (2024), different populations residing in distinct environments, yet with similar ancestry proportions, are influenced by diet, lifestyle, and pathogenic environments, affecting disease prevalence, mortality rates, and longevity [44]. Despite the high frequency of European GA in Brazilian individuals with T1D, Portugal still has a higher European GA than Brazil, which could be related to the higher incidence of T1D in Portugal [10].

Some limitations of the present study must be mentioned: first, it included a smaller number of Portuguese individuals with T1D than those in Brazil. The Brazilian sample is 16 times larger than the Portuguese sample, yet Brazil’s population is approximately 20 times larger than Portugal’s. Second, although our sample was limited to the Central Region of Portugal, studies have shown genetic homogeneity between these regions [45, 49]. Although the Weir & Cockerham F*st* estimator is statistically robust to unequal sample sizes, reduced geographic representation can underestimate within-country diversity in Portugal and potentially inflate between-country F*st* values. Therefore, these comparisons should be interpreted with caution, as part of the observed differences may reflect sampling asymmetry rather than true population-level genetic structure.

A major limitation of this study is the absence of contemporaneous, geographically matched non-T1D controls. Although the Brazilian reference dataset from [20] includes individuals from the same regions as our T1D cohort, it is not temporally matched and therefore cannot be used to infer enrichment or depletion of ancestry associated with disease status. Similarly, while the HGDP-CEPH panel is suitable for defining ancestral clusters, it does not serve as a population control. Thus, our findings allow characterization and comparison of ancestry profiles but do not support causal or etiological interpretations regarding T1D susceptibility. Finally, the use of a 46 AIM-INDEL panel does not allow for optimal refinement of ancestry estimates, and SNP-based approaches would provide more informative resolution of ancestry in contemporary Brazilian populations [13]. In addition, because these markers were specifically selected to maximize intercontinental differentiation, estimates of genetic diversity, FST, and MDS primarily reflect population structure and demographic history, and should not be interpreted as indicators of disease-related genetic risk.

In conclusion, this study reveals differences in GA and diversity among individuals with T1D from Brazil and Portugal. Considering the dynamics of migration and admixture in both countries, our data should inform future research on identifying other genetic variants, such as HLA system alleles (risk or protective) in Brazil and Portugal, that could contribute to a better understanding of the pathogenesis of the disease in both countries.

## Supplementary Information

Additional file 1.

Additional file 2.

## Data Availability

The dataset(s) supporting the conclusions of this article is(are) included within the article (and its additional file(s).

## References

[1] Primavera M, Giannini C, Chiarelli F. Prediction and prevention of type 1 diabetes. Front Endocrinol (Lausanne). 2020;11:248. 10.3389/fendo.2020.00248. 32670194 10.3389/fendo.2020.00248 PMC7326081

[2] Gomes MB, Calliari LE, Conte D, et al. Diabetes-related chronic complications in Brazilian adolescents with type 1 diabetes: a multicenter cross-sectional study. Diabetes Res Clin Pract. 2021;177:108895. 10.1016/j.diabres.2021.108895. 34090967 10.1016/j.diabres.2021.108895

[3] Chiang JL, Kirkman MS, Laffel LMB, Peters AL, Type 1 Diabetes Sourcebook Authors. Type 1 diabetes through the life span: a position statement of the American Diabetes Association. Diabetes Care. 2014;37(7):2034–54. 24935775 10.2337/dc14-1140 PMC5865481

[4] International Diabetes Federation. IDF Diabetes Atlas. 9th ed. Brussels: International Diabetes Federation; 2019.

[5] Diaz-Valencia PA, Bougnères P, Valleron AJ. Covariation of the incidence of type 1 diabetes with country characteristics available in public databases. PLoS One. 2015;10(2):e0118298. 10.1371/journal.pone.0118298. 25706995 10.1371/journal.pone.0118298 PMC4338253

[6] Gomes MB, Rodrigues V, Santos DC, et al. Association between HLA class II alleles/haplotypes and genomic ancestry in Brazilian patients with type 1 diabetes: a nationwide exploratory study. Genes. 2023;14(5):991. 10.3390/genes14050991. 37239351 10.3390/genes14050991 PMC10218425

[7] Ogle GD, James S, Dabelea D, Pihoker C, Svennson J, Maniam J, et al. Global estimates of incidence of type 1 diabetes in children and adolescents: results from the International Diabetes Federation Atlas, 10th edition. Diabetes Res Clin Pract. 2022;183:109083. 10.1016/j.diabres.2021.109083. 34883188 10.1016/j.diabres.2021.109083

[8] International Diabetes Federation. IDF Diabetes Atlas, 11th edn. Brussels, Belgium: 2025. Available at: https://diabetesatlas.org

[9] Zulmira J, Nobre E, Macedo A, et al. Prevalência da diabetes mellitus tipo 1 em Portugal, 1995–1999, coorte de jovens do sexo masculino. Acta Med Port. 2003;16:125–32. 10.20344/amp.1187. 22226211

[10] Portugal. Ministério da Saúde. Direção-Geral da Saúde. Programa Nacional para a Diabetes: Desafios e Estratégias 2024. Lisboa: DGS; 2024.

[11] Negrato CA, Lauris JRP, Saggioro IB, et al. Increasing incidence of type 1 diabetes between 1986 and 2015 in Bauru, Brazil. Diabetes Res Clin Pract. 2017;127:198–204. 10.1016/j.diabres.2017.03.014. 28391136 10.1016/j.diabres.2017.03.014

[12] Vullo C, Gomes V, Romanini C, et al. Association between Y haplogroups and autosomal AIMs reveals intra-population substructure in Bolivian populations. Int J Legal Med. 2015;129(4):673–80. 10.1007/s00414-014-1025-x. 24878616 10.1007/s00414-014-1025-x

[13] Nunes K, et al. Admixture’s impact on Brazilian population evolution and health. Science. 2025;388:eadl3564. 10.1126/science.adl3564. 40373151 10.1126/science.adl3564

[14] Pena SDJ, Di Pietro G, Fuchshuber-Moraes M, et al. Systemic and ocular manifestations of a patient with mosaic <scp>*ARID1A* ‐ </scp> a <scp>ssociated Coffin‐Siris</scp> syndrome and review of select mosaic conditions with ophthalmic manifestations. Am J Med Genet C Semin Med Genet. 2020;184(4):928–38. 10.1002/ajmg.c.31839. 32888375 10.1002/ajmg.c.31839 PMC8808370

[15] Köksal Z, Meyer OL, Andersen JD, et al. Pitfalls and challenges with population assignments of individuals from admixed populations: applying Genogeographer on Brazilian individuals. Forensic Sci Int Genet. 2023;67:102934. 10.1016/j.fsigen.2023.102934. 37713981 10.1016/j.fsigen.2023.102934

[16] Zambrano AK, Gaviria A, Cobos-Navarrete S, et al. The three-hybrid genetic composition of an Ecuadorian population using AIMs-Indels compared with autosomes, mitochondrial DNA and Y chromosome data. Sci Rep. 2019;9(1):9247. 10.1038/s41598-019-45723-w. 31239502 10.1038/s41598-019-45723-w PMC6592923

[17] MacRoberts RA, Liberato M, Roca-Rada X, et al. Shrouded in history: unveiling the ways of life of an early Muslim population in Santarém, Portugal (8th–10th century AD). PLoS One. 2024;19(3):e0299958. 10.1371/journal.pone.0299958. 38446809 10.1371/journal.pone.0299958 PMC10917335

[18] Toso A, Schifano S, Oxborough C, et al. Beyond faith: biomolecular evidence for changing urban economies in multi-faith medieval Portugal. Am J Phys Anthropol. 2021;176(2):208–22. 10.1002/ajpa.24343. 34110625 10.1002/ajpa.24343

[19] MacRoberts RA, Dias CMB, Fernandes TM, Santos AL, et al. Diet and mobility during the Christian conquest of Iberia: a multi-isotopic investigation of a 12th–13th century military order in Évora. Portugal J Archaeol Sci Rep. 2020;30:102210.

[20] Saloum FNM, Pereira R, Vianna R, et al. Revisiting the genetic ancestry of Brazilians using autosomal AIM-indels. PLoS One. 2013;8(9):e75145. 10.1371/journal.pone.0075145. 24073242 10.1371/journal.pone.0075145 PMC3779230

[21] Cavalcante LN, Stefano JT, Machado MV. Genetic ancestry analysis in non-alcoholic fatty liver disease patients from Brazil and Portugal. World J Hepatol. 2015;7(10):1433–8. 10.4254/wjh.v7.i10.1433. 26052389 10.4254/wjh.v7.i10.1433 PMC4450207

[22] Nascimento AF, Oliveira JS, Silva Junior JC, Barbosa AA. Genomic ancestry evaluated by ancestry-informative markers in patients with sickle cell disease. Genet Mol Res. 2016;15(1):gmr15017604. 10.4238/gmr.15017604. 10.4238/gmr.15017604 27051031

[23] Williams RC, Elston RC, Kumar P, et al. Selecting SNPs informative for African, American Indian and European ancestry: application to the Family Investigation of Nephropathy and Diabetes (FIND). BMC Genomics. 2016;17:325. 10.1186/s12864-016-2654-x. 27142425 10.1186/s12864-016-2654-x PMC4855449

[24] Hsu S, Hoofnagle AN, Gupta DK, et al. Race, ancestry, and vitamin D metabolism: the Multi-Ethnic Study of Atherosclerosis. J Clin Endocrinol Metab. 2020;105(12):e4337-50. 10.1210/clinem/dgaa612. 32869845 10.1210/clinem/dgaa612 PMC7526733

[25] Leal DFDVB, da Santana Silva MN, Fernandes DCRO, et al. Amerindian genetic ancestry as a risk factor for tuberculosis in an Amazonian population. PLoS One. 2020;15(7):e0236033. 10.1371/journal.pone.0236033. 32673332 10.1371/journal.pone.0236033 PMC7365596

[26] Kaneva K, Schurr TG, Tatarinova TV, et al. Mitochondrial DNA haplogroup, genetic ancestry, and susceptibility to Ewing sarcoma. Mitochondrion. 2022;67:6–14. 10.1016/j.mito.2022.09.002. 36115539 10.1016/j.mito.2022.09.002 PMC9997094

[27] Rodrigues LM, Maistro S, Katayama MLH, et al. Prevalence of germline variants in Brazilian pancreatic carcinoma patients. Sci Rep. 2024;14(1):21083. 10.1038/s41598-024-71884-4. 39256447 10.1038/s41598-024-71884-4 PMC11387492

[28] Gomes MB, Gabrielli AB, Santos DC, et al. Ethnic differences in antepartum glucose values that predict postpartum dysglycemia and neonatal macrosomia. Diabetes Res Clin Pract. 2018;140:245–52. 10.1016/j.diabres.2018.03.025. 29608977 10.1016/j.diabres.2018.03.025

[29] Pereira R, Phillips C, Pinto N, et al. Straightforward inference of ancestry and admixture proportions through ancestry-informative insertion deletion multiplexing. PLoS One. 2012;7(1):e29684. 10.1371/journal.pone.0029684. 22272242 10.1371/journal.pone.0029684 PMC3260179

[30] Excoffier L, Lischer HE. Arlequin suite ver 3.5: a new series of programs to perform population genetics analyses. Mol Ecol Resour. 2010;10(3):564–7. 10.1111/j.1755-0998. 21565059 10.1111/j.1755-0998.2010.02847.x

[31] Homburger JR, Moreno-Estrada A, Gignoux CR, et al. Genomic insights into the ancestry and demographic history of South America. PLoS Genet. 2015;11(12):e1005602. 10.1371/journal.pgen.1005602. 26636962 10.1371/journal.pgen.1005602 PMC4670080

[32] Balloux F, Lugon-Moulin N. The estimation of population differentiation with microsatellite markers. Mol Ecol. 2002;11(2):155–65. 10.1046/j.0962-1083. 11856418 10.1046/j.0962-1083.2001.01436.x

[33] Pena SDJ, Di Pietro G, Fuchshuber-Moraes M, et al. The genomic ancestry of individuals from different geographical regions of Brazil is more uniform than expected. PLoS ONE. 2011;6(2):e17063. 10.1371/journal.pone.0017063. 21359226 10.1371/journal.pone.0017063 PMC3040205

[34] Gomes MB, Dos Santos GC, de Sousa Azulay RS, et al. <article-title update="added"> Association between <scp>HLA</scp> alleles and haplotypes with age at diagnosis of type 1 diabetes in an admixed Brazilian population: a nationwide study. HLA. 2024;104(1):e15574. 38993161 10.1111/tan.15574

[35] Adams SM, Bosch E, Balaresque PL, et al. The genetic legacy of religious diversity and intolerance: paternal lineages of Christians, Jews, and Muslims in the Iberian Peninsula. Am J Hum Genet. 2008;83(6):725–36. 10.1016/j.ajhg.2008.11.007. 19061982 10.1016/j.ajhg.2008.11.007 PMC2668061

[36] Beleza S, Gusmão L, Lopes A, et al. <article-title update="added">Micro‐phylogeographic and demographic history of Portuguese male lineages. Ann Hum Genet. 2006;70(2):181–94. 16626329 10.1111/j.1529-8817.2005.00221.x

[37] Souza AM, Resende SS, Sousa TN, Brito CFA. A systematic scoping review of the genetic ancestry of the Brazilian population. Genet Mol Biol. 2019;42(3):495–508. 10.1590/1678-4685-GMB-2018-0076. 31188926 10.1590/1678-4685-GMB-2018-0076 PMC6905439

[38] Comini-Frota ER, Brum DG, Kaimen-Maciel DR, et al. Frequency of reported European ancestry among multiple sclerosis patients from four cities in the southern and southeastern regions of Brazil. Clin Neurol Neurosurg. 2013;115(9):1642–6. 10.1016/j.clineuro.2013.02.024. 23535450 10.1016/j.clineuro.2013.02.024

[39] Verdu P, Jewett EM, Pemberton TJ, et al. Expanded satellite repeats amplify a discrete CENP-A nucleosome assembly site on chromosomes that drive in female meiosis. Curr Biol. 2017;27(16):2529–35. 10.1016/j.cub.2017.06.069. 28756949 10.1016/j.cub.2017.06.069 PMC5567862

[40] Korunes KL, Soares-Souza GB, Bobrek K, et al. Sex-biased admixture and assortative mating shape genetic variation and influence demographic inference in admixed Cabo Verdeans. G3 Genes|Genomes|Genetics. 2022;12(10):jkac183. 10.1093/g3journal/jkac183. 35861404 10.1093/g3journal/jkac183 PMC9526050

[41] Pires RP. Portugal: Atlas das Migrações Internacionais. Lisboa: Fundação Calouste Gulbenkian; 2010.

[42] Bryc K, Velez C, Karafet T, et al. Genome-wide patterns of population structure and admixture among Hispanic/Latino populations. Proc Natl Acad Sci U S A. 2010;107(2):8954–61. 10.1073/pnas.0914618107. 20445096 10.1073/pnas.0914618107 PMC3024022

[43] IBGE. Brasil: 500 anos de povoamento. Rio de Janeiro: IBGE; 2000.

[44] Vaulin A, Karpulevich E, Kasianov A, Morozova I. Europeans and Americans of European origin show differences between their biological pathways related to the major histocompatibility complex. Sci Rep. 2024;14(1):21816. 10.1038/s41598-024-71803-7. 39294244 10.1038/s41598-024-71803-7 PMC11410964

[45] Peixoto A, Santos C, Rocha P, et al. The c.156_157insAlu BRCA2 rearrangement accounts for more than one-fourth of deleterious BRCA mutations in northern/central Portugal. Breast Cancer Res Treat. 2009;114(1):31–8. 10.1007/s10549-008-9978-4. 18363094 10.1007/s10549-008-9978-4

[46] StatSoft Inc. STATISTICA (data analysis software system). Version 13. Tulsa (OK): StatSoft; 2024.

[47] SPSS Inc. SPSS for Windows. Version 16.0. Chicago: SPSS Inc.; 2007.

[48] Hout MC, Papesh MH, Goldinger SD. Multidimensional scaling. Wiley Interdiscip Rev Cogn Sci. 2013;4(1):93–103. 10.1002/wcs.1203. 23359318 10.1002/wcs.1203 PMC3555222

[49] Martiniano R, Feitosa Y, Abade A, Manco L. Y-chromosome diversity in central Portugal reveals signatures of ancient maritime expansions. Anthropol Anz. 2013;70(4):355–67. 10.1127/0003-5548/2013/0321. 24620564 10.1127/0003-5548/2013/0321

